# A Python library to check the level of anonymity of a dataset

**DOI:** 10.1038/s41597-022-01894-2

**Published:** 2022-12-26

**Authors:** Judith Sáinz-Pardo Díaz, Álvaro López García

**Affiliations:** Instituto de Física de Cantabria (IFCA), CSIC-UC, Avda. los Castros s/n, 39005 Santander, Spain

**Keywords:** Computer science, Research data

## Abstract

Openly sharing data with sensitive attributes and privacy restrictions is a challenging task. In this document we present the implementation of *pyCANON*, a Python library and command line interface (CLI) to check and assess the level of anonymity of a dataset through some of the most common anonymization techniques: *k-anonymity*, (*α,k)-anonymity*, ℓ*-diversity*, *entropy* ℓ*-diversity*, *recursive* (*c*,ℓ*)-diversity*, *t-closeness*, *basic β-likeness*, *enhanced β-likeness* and *δ-disclosure privacy*. For the case of more than one sensitive attribute, two approaches are proposed for evaluating these techniques. The main strength of this library is to obtain a full report of the parameters that are fulfilled for each of the techniques mentioned above, with the unique requirement of the set of quasi-identifiers and sensitive attributes. The methods implemented are presented together with the attacks they prevent, the description of the library, examples of the different functions’ usage, as well as the impact and the possible applications that can be developed. Finally, some possible aspects to be incorporated in future updates are proposed.

## Introduction

The unstoppable advances in data analysis and processing techniques for knowledge extraction and decision making, whether concerning Big Data or small data, motivate the idea of publishing datasets in an accessible way for the scientific community and society in general. In the same way, the need for collaboration between different institutions, research centers, or even companies, means that they need to be able to share the necessary data among themselves in a secure way. Furthermore, the need to publish open data in order to build an informed society about, for example, the processes of public organizations, intensifies the need to develop tools that allow the publication of such data with privacy guarantees.

When discussing the privacy guarantees that a dataset must fulfill, we are referring to preventing an attacker from extracting sensitive information from a specific individual. There is a strong requirement to protect certain data, such as health records (hospital visits, chronic illnesses), banking information or even police reports. For example, in the case of mental health records, the disclosure of such data about an individual could lead to discrimination and social exclusion. A person’s medical data can also be used in an unethical manner to increase the cost of health insurance, or even lead to social foreclosure if it is revealed that a certain individual suffers from a specific disease. Similarly, information on political beliefs or even on an individual’s monthly income could lead to exclusion and prejudice. Every day we are all producing a digital footprint that reveals our tastes, hobbies or even fears.

A classic example of data that was openly published and eventually showed a security breach is that of the U.S. Census. Specifically, a study conducted on 1990 U.S. Census data revealed that 87% of the time, three pieces of information (zip code, gender and date of birth) were sufficient to identify someone in a database (see^1^). This shows that it is not enough to remove identifiers from a database to prevent an individual from being identified, since he/she can be identified by other types of attributes which a priori seem harmless. Let’s briefly define three key concepts about the attributes or columns of a database. Firstly, the ***identifiers*** are those variables in a database that allow to identify an individual (e.g. name, ID number, email…). On the other hand, the ***quasi-identifiers*** **(*QI)*** are variables that, although a priori seem not to show relevant information, when combined can make possible the identification of an individual, as occurs in the case of the U.S. Census. Moreover, they are accessible to the attacker (e.g. gender, age, city, etc.). Finally, there are the ***sensitive attributes (SA)***, which are the database columns that hold private information that must remain that way and cannot be extracted.

To this aim, in this paper we present *pyCANON*, a Python library and CLI that allows to check if certain anonymity conditions are verified for a dataset, given a set of quasi-identifiers and a set of sensitive attributes. The main idea behind it is to provide researchers, and in general anyone who wants to publish a dataset in open access or to share it with others, with a prior knowledge of the level of anonymization of their data. This will provide insights about the possible risks to which these information would be exposed, allowing users to verify the impact and their resistance to different attacks. An important consideration is that these data have to be provided in tabular format, that is, they cannot be in images, videos or any other kind of unstructured data.

In the following sections, the theoretical bases of the implemented methods are presented, as well as a brief explanation of the main functionalities of the software, examples of use, and the impact and different applications associated with it.

## Results

As explained in the previous section, the purpose of the library presented in this article is, essentially, to check the anonymity level of a dataset. For this purpose, we propose the use of nine different anonymization techniques: *k-anonymity*, *(α,k)-anonymity*, ℓ*-diversity*, *entropy* ℓ*-diversity*, *recursive (c*, ℓ*)-diversity*, *t-closeness*, *basic β-likeness*, *enhanced β-likeness* and *δ-disclosure privacy*. Specifically, given a set of data, a list of quasi-identifiers and a list of sensitive attributes, it will be possible to check for which parameters each of the aforementioned techniques is verified, in order to know the degree of anonymity of such data, and thus the possible risks they may be subject to.

Before exposing the definitions and different aspects taken into account for the implementation of the different techniques, let us present the concept of ***equivalence class (EC)***. An equivalence class is a partition of a database in which all the quasi-identifiers have the same value. That is, users who are in the same equivalence class are all indistinguishable with respect to the quasi-identifiers. The different techniques under study are presented below:

- ***k-anonymity****.* A database verifies *k-anonymity* if each equivalence class of the database has at least *k* rows. In other words, for each row of the database, there are at least *k*-1 indistinguishable rows with respect to the quasi-identifiers. Note that *k*≥1 is always verified and the probability of identifying an individual in the database using the quasi-identifiers will be at most 1/*k*.
- ***(α,k)-anonymity****.* Given only one sensitive attribute *S*, it is checked if the database is *k-anonymous* and the frequency of each possible value of *S* is lower or equal than *α* in each equivalence class.
- ℓ***-diversity****.* In the case of a single sensitive attribute *S*, it is satisfied if for each equivalence class, there are at least ℓ distinct values for *S*. Note that ℓ ≥ 1 is always verified.
- ***Entropy*** ℓ***-diversity****.* A database with a single sensitive attribute *S* verifies this condition if *H*(*EC*) > *log*(ℓ), for every equivalence class *EC* of the database. Note that *H*(*EC*) is the entropy of the equivalence class *EC*, defined as:

$$H(EC)=-\sum _{s\in D}p\left(EC,s\right)\log \left(p\left(EC,s\right)\right),$$

with *D* the domain of *S*, and *p*(*EC*, *s*) the fraction of records in *EC* that have *s* as sensitive attribute.

- ***Recursive (c****,* ℓ***)-diversity****.* The main potential of this technique is that if a value of the sensitive attribute *S* is removed from an equivalence class which verifies *(c*, ℓ*)-diversity*, then *(c*, ℓ*-1)-diversity* is preserved. For the implementation of this technique^2^, has been used as reference (in order to get the formal definition of the concept). Specifically, suppose there are *n* different values for a sensitive attribute *S* in an equivalence class *EC*. Be *r*_*i*_ $(i\in \{1,\ldots ,n\})$ the number of times that the i-th most frequent value of *S* appears in *EC*. Then, *EC* verifies *recursive (c*, ℓ*)-diversity* for *S* if ${r}_{1} < c({r}_{l}+{r}_{l+1}+...+{r}_{n})$. In view of the previous inequality, in the implementation of this technique in *pyCANON*, the value of *c* will not be calculated if a value of ℓ = 1 is obtained.
- ***t-closeness****.* The goal is again similar to that of the two previous techniques. A database with one sensitive attribute *S* verifies *t-closeness* if all the equivalence classes verify it. An equivalence class verifies *t-closeness* if the distribution of the values of *S* are at a distance no closer than *t* from the distribution of the sensitive attribute in the whole database. In order to measure the distance between the distributions, following^3^, the Earth Mover’s distance (EMD) between the two distributions using the ordered distance is applied for numerical sensitive attributes. For categorical attributes, the equal distance is used.
- ***Basic β-likeness and enhanced β-likeness****.* These two techniques have been implemented following the definitions 2 and 3 of^4^, and can be used in order to control the distance between the distribution of a sensitive attribute in an equivalence class and in the entire database. In particular, be ${\mathcal{P}}=\left\{{p}_{1},\ldots ,{p}_{n}\right\}$ the distribution of a sensitive attribute *S* in the whole database and ${\mathcal{Q}}=\left\{{q}_{1},\ldots ,{q}_{n}\right\}$ that of an equivalence class *EC*. Be $max(D({\mathcal{P}},{\mathcal{Q}}))=max\left\{D({p}_{i},{q}_{i}):{p}_{i}\in {\mathcal{P}},{q}_{i}\in {\mathcal{Q}}\wedge {p}_{i} < {q}_{i}\right\}$, then *basic β-likeness* is verified if $max(D({\mathcal{P}},{\mathcal{Q}}))\le \beta$ and *enhanced β-likeness* is verified if $D({p}_{i},{q}_{i})\le min\left\{\beta ,-\log ({p}_{i})\right\}$ $\forall {q}_{i}\in {\mathcal{Q}}$. In both cases *β* > 0. Note that *enhanced β-likeness* provides more robust privacy than *basic β-likeness*.

In the implementation of this tool in *pyCANON* the relative distance function is considered in order to calculate the distance between the distributions, that is: $D\left({p}_{i},{q}_{i}\right)=\frac{{q}_{i}-{p}_{i}}{{p}_{i}}$.

- *δ****-disclosure privacy****.* Considering a database with only one sensitive attribute *S*, be *p*(*EC*, *s*) the fraction of records with *s* as sensitive attribute in the equivalence class EC, *p*(*DB*, *s*) that for the whole database (DB). Then, *δ-disclosure privacy* is verified iff:

$$\left|\log \left(\frac{p(EC,s)}{p(DB,s)}\right)\right| < \delta ,$$

for every $s\in D$ (with *D* the domain of *S*) and every equivalence class *EC*^5^.

The motivation for including the nine techniques outlined above and not a smaller number of them or just the most classic ones (e.g. *k-anonymity* or ℓ*-diversity*), is that they are not all useful against the same types of attacks. That is, there are techniques that are very useful against certain attacks but cannot prevent from others. In particular, Table 1 briefly describes some of the most common attacks which databases can suffer, namely: *linkage*, *re-identification*, *homogeneity*, *background knowledge*, *skewness*, *similarity* and *inference attacks*.

**Table 1:** Common attacks on databases and description.

Attack	Description
*Linkage*	Consists of combining at least two anonymized databases in order to reveal the identity of some individuals present in both.
*Re-identification*	This kind of attacks occurs when the anonymization process is reversed.
*Homogeneity*	Can occur when all the values for a sensitive attribute in an equivalence class are identical.
*Background knowledge*	In this case, the adversary has some foreknowledge about the target of the attack (e.g. knows some auxiliary information about an individual in the database).
*Skewness*	Can be carried out when there is an unfrequent value for a sensitive attribute in the whole database which is extremely frequent in an equivalence class.
*Similarity*	May occur when the values of a sensitive attribute in an equivalence class are semantically similar (although different).
*Inference*	Consists of using data mining techniques in order to extract information from the data.

In addition, Table 2 shows the most convenient techniques (although not the only ones) that can be applied to prevent each of the previously mentioned attacks.

**Table 2:** Anonymization techniques and principal attacks that prevent (among others).

*Technique*	Principal attack which prevents						
	Linkage	Re-identification	Homogeneity	Background	Skewness	Similarity	Inference
*k-anonymity*	✓	✓					
*(α,k)-anonymity*	✓	✓	✓				
ℓ*-diversity*			✓	✓			
*Entropy* ℓ*-diversity*			✓	✓			
*Recursive (c*, ℓ*)-diversity*			✓	✓			
*t-closeness*					✓	✓	
*Basic β-likeness*					✓		
*Enhanced β-likeness*					✓		
*δ-disclosure privacy*					✓		✓

It is important to take into account that the values of *t* and *δ* for *t-closeness* and *δ-disclosure privacy* respectively must be strictly greater than the ones obtained using *pyCANON* (see the definition of that technique).

On the other hand, it should be noted that although the anonymization techniques have been presented for the case where the database consists of a single sensitive attribute, they can nevertheless be applied in the case of multiple sensitive attributes. The latter may be quite common since in many use cases there are several attributes deemed to be sensitive information. Specifically, this library implements two approaches that can be followed in this case:

1. In the simplest case, for each sensitive attribute (SA) each of the properties is checked and the parameter that is satisfied for all of them is kept (e.g. for ℓ*-diversity* the smallest value of ℓ once it is computed for each SA will be kept, while the value of *α* for *(α,k)-anonymity* will be the largest value of *α* of those obtained for each SA). In the following we will refer to this approach as the *harmonization of quasi-identifiers* one (or simply *harmonization*).
2. In the second approach, we will have to update the set of quasi-identifiers according to the sensitive attribute to be analyzed, and proceed again as in the previous case. That is, be *Q* initial of the set of quasi-identifiers, and *S* the set of sensitive attributes. For each sensitive attribute ${S}_{i}\in S$, the set of quasi-identifiers considered in each case would change (and with it the different equivalence classes), so that it would be $Q\cup (S\backslash {S}_{i})$. In the following we will refer to this approach as that of the *quasi-identifiers update*. The purpose of this second strategy is to address the possibility that an attacker may be aware of some of the sensitive attributes, acting as quasi-identifiers that would enable inferences about the remaining sensitive information.

As will be explained in the following, *pyCANON* implements the two previously exposed approaches, and it is up to the user to select which one to use. One of the main points to take into account is that the second approach, in which the set of quasi-identifiers is updated according to the sensitive attribute to be analyzed, is more computationally expensive, since for each sensitive attribute the equivalence classes must be recalculated (because the set of quasi-identifiers varies, in addition to being larger). In the case where the data to be checked for anonymity level have been previously anonymized according to the sensitive attributes, the user may choose to check the level with both approaches in case he/she does not know which approach has been followed, and consider the most suitable one for the particular purpose.

The different functions available in this framework are shown in the following section, together with some use examples, but first, let’s briefly review the related work in the area and other anonymization tools that are worthy of special mention.

### Related work

In order to provide users with tools to control the level of anonymity of the data prior to their publication or use in a data science pipeline, several software projects and libraries emerge. The main tool to highlight when talking about data anonymization is the ARX Software^6^: ARX is a comprehensive open source software for anonymization of sensitive data that supports a wide variety of privacy, risk and quality models, together with methods for data transformation and for analyzing the usefulness of the output data. PRIVAaaS^7^ is another tool focused on providing policy-based anonymization in distributed data processing environments, aimed at reducing the data leakage in Big Data processing. ARGUS (including the *μ*-ARGUS and *τ*-ARGUS packages) is a software library for Statistical Disclosure Control delivered by the CASC-project focused on microdata and tabular data. Other tools worth highlighting are *Docbyte’s tool for real-time automated anonymization* (https://www.docbyte.com/anonymization/), which uses machine learning techniques and can be applied for blur or black out images, and *g9 Anonymizer* (https://www.esito.no/en/products/anonymizer/), which is a tool for data anonymization that also aligns with GDPR principles, e.g. by supporting data portability and access rights.

As presented in^8^, the desired level of anonymity is mostly achieved by applying hierarchies and generalization, as well as by suppressing records. For example, in^9^ a framework for efficient privacy preservation (in particular through *k-anonymity* and ℓ*-diversity*) is presented, starting with the case of one-dimensional quasi-identifiers, and then mapping to the multi-dimensional case using meaningful information loss metrics. Also worth mentioning is the study conducted in^10^ where a review of anonymization methods for parallel stream data is presented together with a very interesting analysis of related work in the area.

In addition, due to the importance of anonymizing sensitive information in different fields of science, it is also relevant to point out the work done for example in^11^, where a data anonymization pipeline to promote Open Science in the context of COVID-19 is presented. Furthermore^12^, presents a methodology for the pseudonymization in the field of medical data in order to prevent data disclosure.

## Discussion

As already stated, the purpose of *pyCANON* library is to assess the level of anonymity of a dataset with regards to the most common techniques, being complementary to other anonymization tools like ARX. In this section, a battery of applications that validate the impact and usefulness of this library for different purposes is discussed. In particular, different examples are presented using openly available data, so that the reproducibility of the results is ensured. However, note that as will be explained later, many of these data have been anonymized using the open source software *ARX*^6^, establishing different levels of hierarchies for the generalization of the quasi-identifiers, which could cause the results to vary according to the different hierarchies introduced.

Suppose we have a dataset and a list of quasi-identifiers for which we want to get the data to verify *k-anonymity* with, for instance, *k* = 5. To do this, we can use a software like *ARX*, selecting as quasi-identifiers the columns of the data set that interest us. Suppose that by mistake, one of the quasi-identifiers is introduced as an insensitive attribute in the anonymization process (for example, in *ARX* there is a drop-down to distinguish the type of attribute, so it is not complex to imagine a possible human error). Once anonymized using this software, we obtain a dataset verifying *k-anonymity* with *k* = 5 for the columns included as quasi-identifiers. Then, *pyCANON* can be used to check if the new dataset truly verifies *k-anonymity* for *k* = 5 for the initial given list of quasi-identifiers. However, suppose that by doing this check we get a value *k* = 1, i.e., there is at least one set of quasi-identifiers which only has a single individual (an equivalence class with only one row). This allows us to quickly detect that there has been a failure in the anonymization process (in this case, at least one of the columns that should be a QI, was introduced by mistake in *ARX* as insensitive value). In this way, by checking the anonymity level with *pyCANON* we have managed to prevent a bug that can be very common, but that would allow an attacker to extract unwanted information about the dataset.

Another example of using the library will be now presented with the stroke dataset^13^, which contains information on 5110 patients with the following set of quasi-identifiers (QI) and sensitive attributes (SA): **QI** **=** **[‘gender’, ‘age’, ‘hypertension’, ‘heart_disease’, ‘ever_married’, ‘work_type’, ‘Residence_type’, ‘smoking_status’]** and **SA** **=** **[‘stroke’]**. Again, using *ARX k-anonymity* will be applied for different values of *k*, namely *k* = 2, 5, 10, 20, 25. Note that the sensitive attribute is discrete and only can take two different values. For the quasi-identifiers, in the case of *age* (which takes values between 1 and 82) 6 levels of hierarchies have been included, starting with intervals of 4 years, being the first one [1, 5), [5, 9), …, [77, 81), [81, 83), the fifth level [1, 65), [65, 83), and the last one including all the possible values. For the remaining *QI*, since all of them are of binary type except *work type* (only takes 5 possible values) and *sex* (which takes three possible values: male, female and other), only the possibility of suppressing the value by replacing it by * is introduced. Then, for example by setting *k* = 20 we can check with *pyCANON* that the actual *k* value obtained for the resulting dataset is *k* = 22. This does not mean that the anonymization done with *ARX* is wrong, because if *k-anonymity* is verified for *k* = 22, it is evidently verified for *k* = 20. In fact the software itself warns us of this if, because in the properties of the data it indicates that the size of the smallest equivalence class is 22. This can be due because with the entered hierarchies, the greater value of *k* which verifies *k-anonymity* with *k* = 20, is 22. Similarly, if in this same example we set *k* = 25 in *ARX*, then we can check with *pyCANON* that the maximum value for *k* is 27.

Another practical example is the following: suppose we have a dataset to which *k-anonymity* has been applied for a certain value of *k*, again, for example, using *ARX*. We may be interested, without applying any further technique, to check some of the other previously exposed anonymization techniques and compare how these scale as a function of the value of *k*. For example, let’s consider the drug classification dataset^14^, composed by 200 records and with the following sets of quasi-identifiers and sensitive attributes: **QI** **=** **[‘Age’, ‘Sex’, ‘BP’, ‘Cholesterol’, ‘Na_to_K’]** and **SA** **=** **[‘Drug’]**. These data have been anonymized using *ARX* only considering *k-anonymity* for different values of *k*, namely *k* = 2, 5, 10, 15, 20, including only hierarchies to generalize on two variables, *Age* (with 3 levels of intervals starting with [15, 25], …, [65, 75]) and *Na to K* (with 5 levels of hierarchies based on intervals). Let’s see in Table 3 how to scale, for example, the values of ℓ for ℓ*-diversity*, *t* for *t-closeness*, and *β* for *basic β-likeness* (without removing the values suppressed when anonymizing with *ARX*).

**Table 3:** Values of ℓ for ℓ*-diversity*, *t* for *t-closeness*, and *β* for *basic β-likeness* obtained for a prefixed value of *k* for *k-anonymity* considering the drug dataset.

*k (k-anonymity)*	ℓ *(*ℓ*-diversity)*	*t (t-closeness)*	*β (basic β-likeness)*
**2**	1	0.93	11.5
**5**	2	0.68	8.38
**10**	2	0.63	5.62
15*	2	0.48	5.62
20**	2	0.38	2.32

*k = 15 has been set with *ARX*, and can be checked with *pyCANON* that the resulting data also verify *k-anonymity* for *k* = 17.

**k = 20 has been set with *ARX*, and can be checked with *pyCANON* that the resulting data also verify *k-anonymity* for *k* = 21.

Thus, as can be seen in Table 3, it is enough to apply the *k-anonymity* process (which requires little computational cost and, depending on the size of the dataset, in *ARX* is done in a few seconds), to obtain better values for the parameters of other anonymization techniques, such as increasing the value of ℓ for ℓ*-diversity*, or decreasing the value of *t* for *t-closeness*. It can also be applied to a dataset that has not been previously processed, to check which techniques should be applied, which are the most significant quasi-identifiers (that provide more information), etc. For example, given an unprocessed dataset, the values of *k* and ℓ obtained with the different permutations of the quasi-identifiers could be calculated, in order to evaluate the possibility of eliminating some of them (cataloging them as sensitive attributes for instance).

The same has been done again for the case of the adult database^15^, considering a subsample of 32561 records. Specifically, six quasi-identifiers (QI) and one binary sensitive attribute (SA) have been chosen: **QI** **=** **[‘age’, ‘education’, ‘occupation’, ‘relationship’, ‘sex’, ‘native-country’]** and **SA** **=** **[‘salary-class’]**. The sensitive attribute (*salary class*) only takes two possible values: 50 *K* or ≤50 *K*, depending on whether the individual’s salary exceeds or not 50000$. Note that for the cases of *sex* and *relationship* no hierarchies are applied, since the former takes only two possible values, and the latter only six. While for these two attributes it is only allowed to generalize by substituting the value of *, the following hierarchical levels are considered for the rest of the quasi-identifiers:

- *age:* five levels of hierarchies are applied, starting with 5-year intervals, followed by 10-year intervals, 20-year intervals, 40-year intervals and finally the case [0, 80) or + 80 at the last level.
- *education:* two levels of hierarchies are applied. In the first one the possible values are: *Primary School*, *High School*, *Undergraduate*, *Professional Education* and *Graduate*. In the second level a further generalization is made, including only three possible values: *Primary education*, *Secondary education* and *Higher education*.
- *occupation:* a hierarchy encompassing the different options in three categories is included, namely: *technical*, *non-technical* and *other*.
- *native country:* the hierarchy simply generalizes the country according to the continent. The value *unknown* is introduced if the value of the native country field was?

**Fig. 1:**
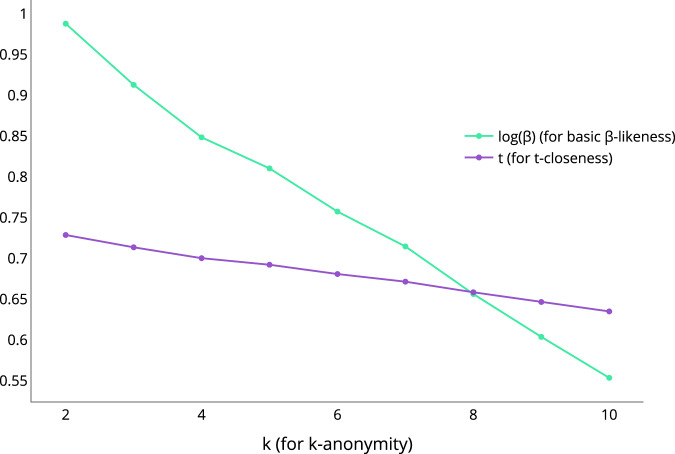
Evolution of *t* for *t-closeness* and log(*β*) for *basic β-likeness* when varying the value of *k* for *k-anonymity*.

Specifically, the adult dataset has been anonymized using *ARX* considering all $k\in [2,10]\cap {\mathbb{N}}$ for *k-anonymity* and the hierarchies exposed above. Figure 1 shows the evolution of the values of *t* and *β* (the latter is shown in logarithmic scale for better visualization) for *t-closeness* and *basic β-likeness* respectively, removing the values suppressed by *ARX*. A substantial decrease is produced both for values of *t* and *β* (remember that *t* is strictly greater than the value obtained using *pyCANON*).

Finally, let us see the results obtained with *pyCANON* for three different techniques of the nine described above after anonymizing using the *ARX* Software. The adult dataset will be used with the quasi-identifiers, sensitive attributes and hierarchies presented in the previous example. With this analysis we want to show the usefulness of this library to check that an anonymity process has been carried out correctly. Tables 4, 5 show the results obtained by setting certain values of *t* and *δ* for *t-closeness* and *δ-disclosure* respectively in *ARX*, and those obtained using *pyCANON* (without removing the records suppressed when anonymizing with *ARX*).

**Table 4:** Values for *t-closeness* using *ARX* and *pyCANON*.

*t for t-closeness ARX*	*t for t-closeness pyCANON*
0.1	0.09795
0.05	0.04773
0.04	0.03484
0.03	0.02828
0.02	0.01550
0.01	0.00919

**Table 5:** Values for *δ-disclosure* using *ARX* and *pyCANON*.

*δ for δ-disclosure ARX*	*δ for δ-disclosure pyCANON*
0.1	0.06237
0.05	0.03339
0.04	0.01661
0.03	0.01661
0.02	0.01352
0.01	0.00293

It should be noted that in the case of *basic β-likeness*, differences may occur depending on the function used to calculate the distance. In the case of *pyCANON*, the relative distance is implemented. However, Table 6 shows five examples where the values established with *ARX* and the results with *pyCANON* are compared. The same will occur with other methods depending on the strategy or definition considered in each case, as they may differ.

**Table 6:** Values for basic *β*-likeness using ARX and pyCANON.

*β for basic β-likeness ARX*	*β for basic β-likeness pyCANON*
0.1	0.09766
0.05	0.04909
0.01	0.00523
0.005	0.00425
0.001	0.00093

In addition, several test have been carried out for the value of *k* for *k-anonymity*, using five different datasets. All of them can be found in the folder *examples* of the original framework repository. In these examples, a case with several sensitive attributes is included, and the values obtained with each of the two proposed approaches are studied.

All these functionalities will allow researchers and users in general to improve their practices regarding the publication of data using anonymization techniques, thus helping to raise awareness of the importance of the anonymization process. That is, it will enable data to be published with greater security guarantees. Again, if a database has been anonymized with a particular software, it would not be very appropriate to check the anonymization level with it, because if there is a bug, it would not be detected when checking. However, *pyCANON* is a library that allows to check the correct application of these techniques, as well as to test for which parameters those techniques that have not been consciously applied would verify with. This would change the daily practices of those who are in charge of publishing data, but also for those institutions, centers or researchers who collaborate by sharing data between them, because they could be sure of the privacy level of the information which they are going to share.

It is important to remark that it is desirable that the data under study have previously undergone some transformation before using *pyCANON*, otherwise the size of the equivalence classes are likely to be very small and hence the computational cost of calculating them will be high. In addition, it is noteworthy that certain techniques acting on sensitive attributes may be more restrictive than others, for instance *entropy* ℓ*-diversity* is more restrictive than *l-diversity* or *δ-disclosure privacy* and as its name indicates, the value obtained for *enhanced β-likeness* is more restrictive than one obtained with *basic β-likeness*.

Finally, highlight that one of the main strengths of this library is that it does not require strong knowledge of Python language, since it is designed so that the user only has to enter a *pandas* dataframe with the data, a string list with the names of the columns that are quasi-identifiers, and another with those that are sensitive attributes. This makes it a library accessible to the general public wishing to check the anonymity level of a dataset regardless of its background.

## Methods

In this library, the user is provided with a set of functions to calculate the anonymity level of a dataset based on the techniques mentioned previously. In addition, for the case of multiple sensitive attributes, it allows to know the results for the two approaches previously exposed, namely: *harmonization* or *quasi-identifiers update*.

Specifically, a function for the calculation of each of the anonymity techniques presented has been implemented in the package *anonymity*, being *data* a *pandas* dataframe containing the dataset under study. Be *qi* the list of quasi-identifiers and *sa* that of the sensitive attributes. The parameter *gen* indicates with approach should be applied in case of multiple sensitive attributes: If *True* (default) harmonization approach is applied, if *False*, the set of quasi-identifiers is updated for each sensitive attribute. Then, the different functions used to calculate the parameters for the previously mentioned properties are exposed in Table 7. Remember that the values of *t* and *δ* for *t-closeness* and *δ-disclosure privacy* must be strictly greater than the ones obtained using the functions of *pyCANON*.

**Table 7:** *pyCANON* main functions to check anonymity properties of a dataset.

Parameter calculated	Function
*k* for *k-anonymity*	*k_anonymity(data, qi)*
*α* and *k* for *(α, k)-anonymity*	*alpha_k_anonymity(data, qi, sa, gen)*
ℓ for ℓ*-diversity*	*l_diversity(data, qi, sa, gen)*
ℓ for *entropy* ℓ*-diversity*	*entropy_l_diversity(data, qi, sa, gen)*
*c* and ℓ for *recursive (c*, ℓ*)-diversity*	*recursive_c_l_diversity(data, qi, sa, gen)*
*β* for *basic β-likeness*	*basic_beta_likeness(data, qi, sa, gen)*
*β* for *enhanced β-likeness*	*enhanced_beta_likeness(data, qi, sa, gen)*
*t* for *t-closeness*	*t_closeness(data, qi, sa, gen)*
*δ* for *δ-disclosure privacy*	*delta_disclosure(data, qi, sa, gen)*

**Fig. 2:**
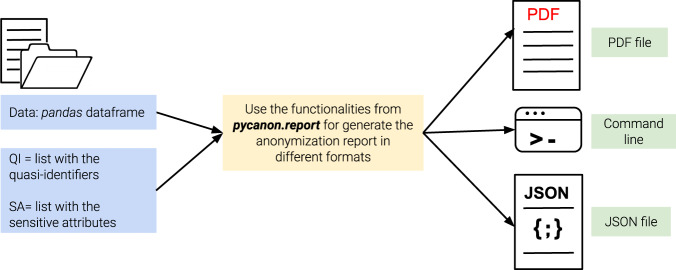
Schema for obtaining the anonymization report from the data, list of quasi-identifiers and sensitive attributes.

In addition, the package *report* is a key functionality of this library. Again *data*, *qi*, *sa* and *gen* are defined as in the previous example. The purpose of this package is to generate a report with the anonymization level of the data file entered, checking all the techniques mentioned above. This report can be generated into a *JSON* file, a *PDF* file, or displayed on the screen. An example will be shown in the following *Usage examples* Subsection, but the basic schema for using this package is represented in Fig. 2.

Besides being a library with the two packages mentioned above: *anonymity* and *report*, *pyCANON* is also implemented as a command line interface (CLI). The latter allows the user to execute the code via a simple command line with interactive use via text input.

Finally, it is important to note that the overall structure of the library includes, among others, unit tests, documentation, and files with example data as well as their corresponding tests. Also, note that the documentation of the framework can be found at https://readthedocs.org/projects/pycanon/. A schematic overview of the structure of the library and a brief outline of the implementation is given in the following.

### Library structure

The following is an outline of the global structure of the library. Note that in the *pycanon* folder the two packages already mentioned above are included: *anonymity* and *report*. Note that a folder with usage examples is provided (*examples*) and another one containing both raw, anonymized and processed data (*data*). In order to ensure that the code quality standards are met, several unit tests have been carried out, which can be found in the *tests* folder.

For more information, the contents of the *anonymity* package along with the subpackage *utils* and the implemented functions are listed at https://pycanon.readthedocs.io/en/latest/pycanon.anonymity.html and that of the *report* package at https://pycanon.readthedocs.io/en/latest/pycanon.report.html.
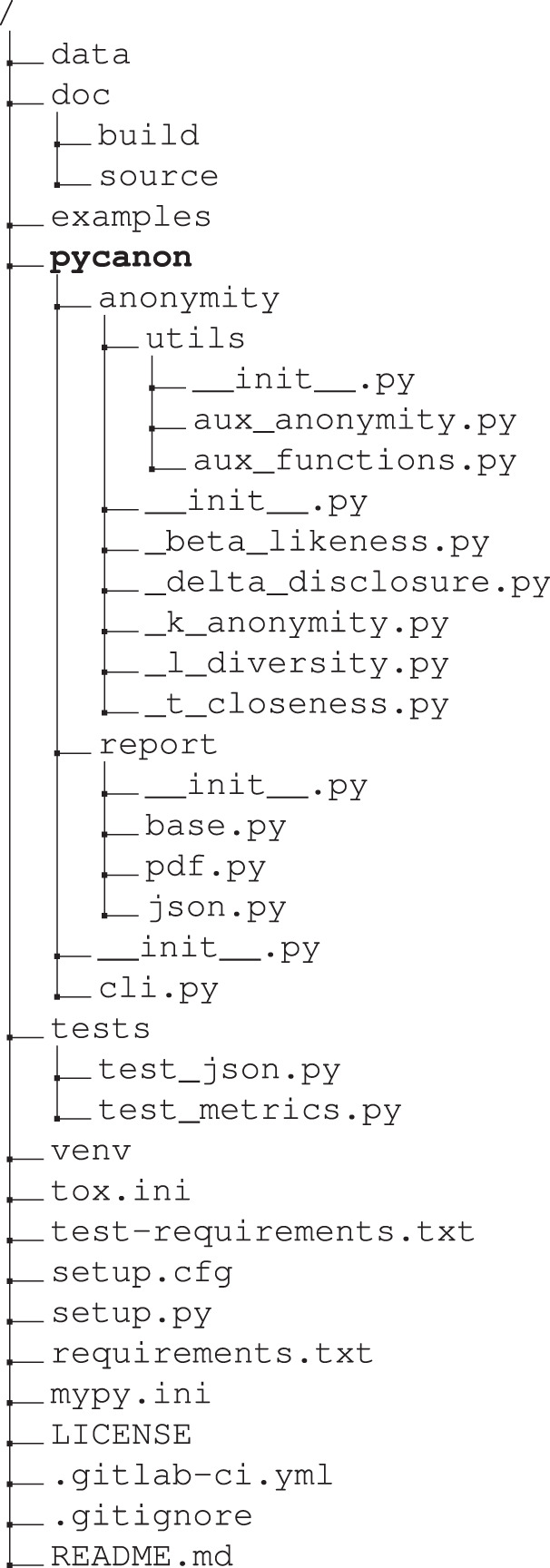


### Details on the implementation

Now, let’s briefly discuss some details about the implementation of the different anonymity techniques that compose the *pyCANON anonymity* package. Note that for more details on the implementation all the code is available in the library repository https://github.com/IFCA/pycanon. In particular, with the exception of *k-anonymity* which does not depend on the set of sensitive attributes, the other eight techniques call an auxiliary function acting on a single sensitive attribute and a given set of quasi-identifiers. Thus, depending on the approach taken by the user in the case of more than one sensitive attribute (*harmonization* if *gen* = *True* or *quasi-identifiers update* if *gen* = *False*), the auxiliary function will be called for one set of quasi-identifiers or another as exposed when presenting two approaches. In all the auxiliary functions and in the function for the *k-anonymity* calculation, it is necessary to obtain the equivalence classes in terms of the entered quasi-identifiers. For this purpose, we show the implemented code in the Example Code 1.
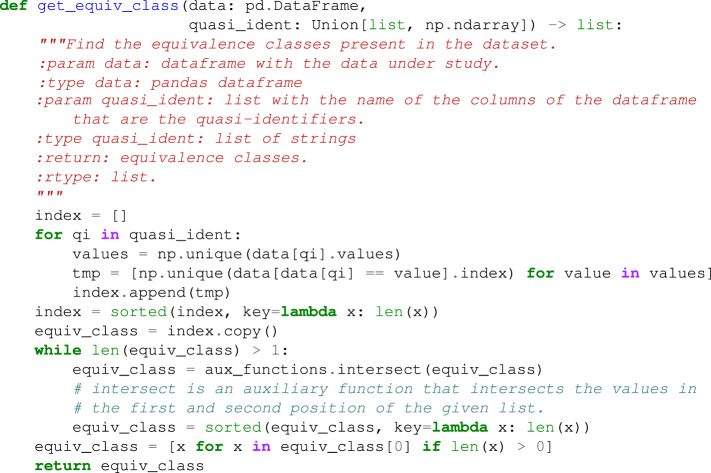


### Example Code 1

**Fig. 3:**
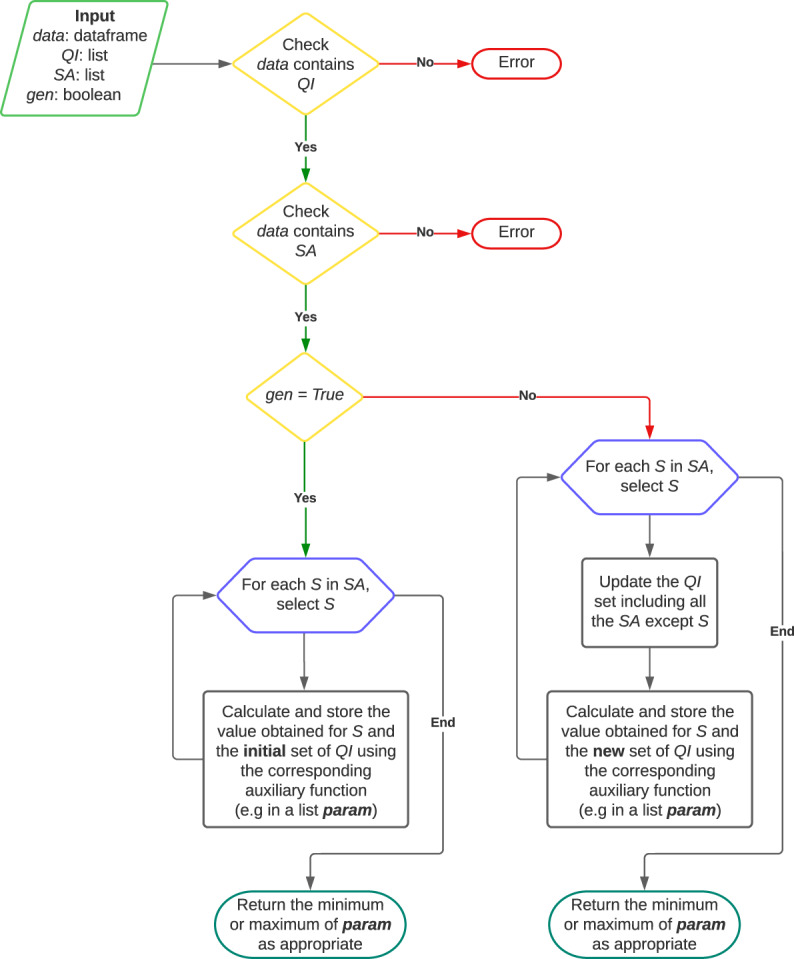
Flux for the implementation of the eight methods presented in *pyCANON* which use the sensitive attributes.

Function to compute the equivalence classes of a dataset given a list of quasi-identifiers. In the case of *k-anonymity*, it is obvious that in order to find the value of *k* the procedure is as simple as taking the minimum of the length of each equivalence class. For the rest of the functions, the definition presented for each of them in the *Results* section will be applied. The flow to be followed in the case of several sensitive attributes is given in Fig. 3. In this figure, the input contains the pandas dataframe with the data, a list with the quasi-identifiers (QI), a list with the sensitive attributes (SA) and a boolean (*gen*) specifying the approach to be followed in the case of more than one sensitive attribute. In addition, in the last step represented in the flow of Fig. 3, when we say “return the minimum or maximum of *param* as appropriate”, we mean that, for example, in the case of ℓ*-diversity* we should return the minimum while in the case of *t-closeness*, the maximum. As already mentioned, for each of the eight techniques using the sensitive attributes there is an auxiliary function that calculates the parameter in question for a single sensitive attribute.

### Usage examples

Let us start by presenting the library usage by means of a the already presented adult dataset, widely used in the field of data privacy, anonymization and pseudoanonymization. Specifically, the same quasi-identifiers (QI), sensitive attribute (SA) and hierarchies considered in the *Discussion* section have been chosen: **QI** **=** **[‘age’, ‘education’, ‘occupation’, ‘relationship’, ‘sex’, ‘native-country’]** and **SA** **=** **[‘salary-class’]**. As already stated, it is highly recommended to anonymize the data before using *pyCANON* according to some technique (e.g. *k-anonymity*) since otherwise the computational cost will be excessively high due to the large number of equivalence classes in the raw data. In this example we are considering the data obtained after applying *k-anonymity* with *k* = 3, stored in *adult_anonymized_3.csv* and available in the folder *data/processed* of *pyCANON* repository. *FILE_NAME* is a string with the path to the *.csv* file where the mentioned dataset is stored (in this case *FILE_NAME* = *‘adult_anonymized_3.csv’*). First, we must import the package *anonymity* from the *pyCANON* library and then run a function for each of the properties studied above, as follows:
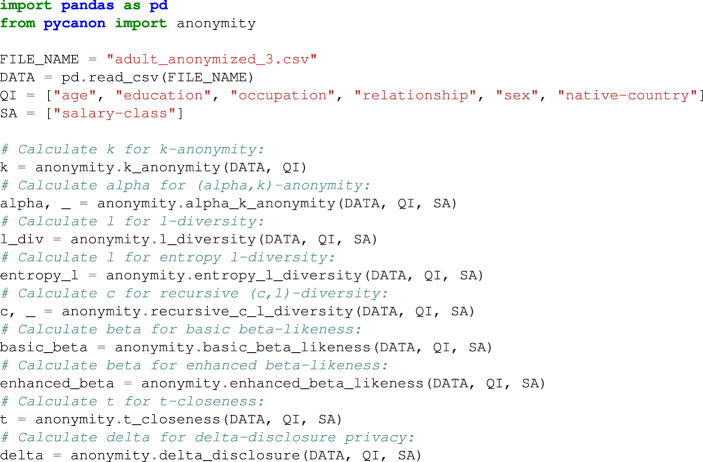


### Example Code 2

Use example of the *anonymity* package.

The above example can be easily reproduced by simply installing the library, downloading the aforementioned anonymized dataset (available in the folder *data/processed* of *pyCANON* repository), reading it with pandas and applying the different functions from *pycanon.anonymity*.

Next, it is interesting to highlight how to proceed in the case of multiple sensitive attributes. The process is again very simple, but first the approach to be followed must be selected: *harmonization of quasi-identifiers* or *quasi-identifiers update*. In the first case, the procedure to follow is the same as the previous example, where the different functions were called for the case of a single sensitive attribute, but now, introducing two or more values in the list **SA**. By default the configuration of the functions selects the harmonization approach if it detects more than one sensitive attribute. However, if one wants to use the approach of updating the quasi-identifiers (which is stricter from a privacy point of view), in all those functions where the list of sensitive attributes (**SA** in the example above) is entered, it is necessary to add a last attribute, *gen* = *False*. This is indicating in each function that the conditions must be checked by updating the set of quasi-identifiers for each sensitive attribute under study.

Regarding the process to obtain the anonymization report, the process to follow is simple. If one wants to save the report as a *PDF* file, use the module *pdf* from *pycanon.report*, and just indicate the path to the PDF file where it is to be saved in the variable *FILE_PDF*, together with the data, quasi-identifiers, sensitive attributes and the value for the parameter *gen*. In the same way, if one wants to save the report as a *JSON* file, use the module *json* from *pycanon.report*. Note that it is also possible to display the report by command line using the function *print_report*(*)* from the package *report*. In the following, an example of displaying the report in a PDF file is shown.
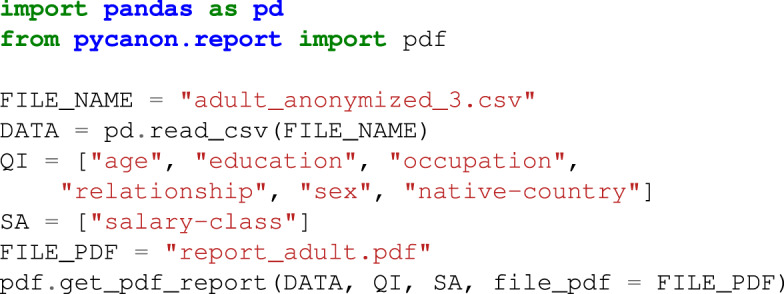


### Example Code 3

Use example of the report package

### Requirements

For the installation and use of *pyCANON*, we recommend using a virtual environment with Python 3.8 or higher. Specifically, the installation of *pyCANON* (version 1.0.0) can be performed using the package-management system *pip*, namely pip install pycanon. The additional libraries required are included in the *requirements.txt* file in the library repository and are the following: *numpy* (version ≥ 1.21.6, https://numpy.org/), *pandas* (version ≥ 1.3.5, https://pandas.pydata.org/), *reportlab* (version ≥ 3.6.9, https://www.reportlab.com/), *typer* (version ≥ 0.4.1, https://typer.tiangolo.com/) and *tabulate* (version ≥ 0.8.10, https://github.com/astanin/python-tabulate).

### Conclusions and future work

In the previous sections nine anonymization techniques have been presented, from the most classical ones (e.g. *k-anonymity* and ℓ*-diversity*) to other less common ones (e.g. recursive (*c*, ℓ*)-diversity*, *enhanced β-likeness* or *δ-disclosure privacy* among others). The implementation of *pyCANON*, a Python library and CLI for checking these anonymity properties is presented. The objective is the following: given a data set, a list of quasi-identifiers and a list of sensitive attributes, to evaluate for which parameters these techniques are satisfied. In addition, two different approaches to follow in case of multiple sensitive attributes are presented: *harmonization of quasi-identifiers* and *quasi-identifiers update*.

Different useful applications of this framework are discussed throughout this work, from checking if errors have occurred in the anonymization process (this can be very important, for example, before making the data public) to, among others, seeing how different properties scale as a function of others. Specifically, in Fig. 1 an example of how the values of *t* for *t-closeness* and *β* for *basic β-likeness* evolve as *k* increases for *k-anonymity* is presented. In this same line Table 3 shows the evolution of ℓ, *t* and *β* for ℓ*-diversity*, *t-closeness* and *basic β-likeness* respectively when varying *k* for *k-anonymity*. On the other hand, another example shown is the case in which due to the hierarchy levels established it can be verified that although a certain value *k* has been established for *k-anonymity*, it can be verified that in effect this property is verified for another value *k*′ > *k*.

In the first release of this library, nine techniques exposed when presenting the methods have been included, but this software may be extended in future versions with new functionalities. Among them, the idea of applying techniques such as *δ-presence* or *k-map*, for which it is necessary to use an auxiliary population, seems really attractive, along with others such as *m-invariance*, which can be used in order to preserve privacy when re-publishing datasets^16^. In addition, it is also proposed for future updates and improvements of the library to include, together with the anonymity report that can be generated using the *report* package, personalized recommendations on the values obtained: for example, to inform that it is advisable to have a value of ℓ for ℓ*-diversity* strictly greater than one (and ℓ = 2 if the sensitive attribute is binary) or that is recommended for the value of *α* for (*α, k)-anonymity* to be strictly lower than one. Furthermore, these recommendations could be customized according to the number of data, as well as the quasi-identifiers and sensitive attributes involved.

Moreover, it is also very interesting the analysis of other techniques clearly on the rise in the field of data privacy, such as *ε-differential privacy* and (*ε*, *δ*)*-differential privacy*^17^.

Finally, in addition to knowing the level of privacy of data before publication or before using them in an inference process, for example through the use of machine learning techniques, it is of critical importance to know the usefulness of the data after anonymization. To this end, as a future work the implementation of a new module to extend *pyCANON* is proposed. Specifically, the idea would be to introduce the original raw dataset (without undergoing any transformation) together with the anonymized one in order the calculate the utility. The objective will be for *pyCANON* to report the level of anonymity with respect to the methods consulted by the user, together with the utility level of these data based on different metrics.

## Data Availability

No new data was generated in this work.

## Code availability

Code for the *pyCANON* library and each compliance test is available in https://github.com/IFCA/pycanon. The library documentation can be found in https://pycanon.readthedocs.org.
